# Higher Levels of *BRCA1* Gene Methylation in Sporadic Breast Cancer Patients with a Lower Incidence of Recurrence

**DOI:** 10.3390/medsci14020251

**Published:** 2026-05-13

**Authors:** Grasiela Agnes, Andrea Pires Souto Damin, Guilherme Watte, Giuliano Rizzotto Guimarães, Adriana Vial Roehe, Jenifer Saffi

**Affiliations:** 1 Laboratory of Molecular Biology, Federal University of Health Sciences of Porto Alegre (UFCSPA), Rua Sarmento Leite, 245, Porto Alegre 90050-170, RS, Brazil; 2 Division of Breast Surgery, Hospital de Clinicas de Porto Alegre, Federal University of Rio Grande do Sul (UFRGS), Porto Alegre 90035-903, RS, Brazil; adamin@hcpa.edu.br; 3 Epidemiology, Department of Respiratory Medicine and Thoracic Surgery, Irmandade Santa Casa de Misericórdia de Porto Alegre, Porto Alegre 90020-090, RS, Brazil; guilherme.watte@ufcspa.edu.br; 4 Laboratory of Pathology, Federal University of Health Sciences of Porto Alegre (UFCSPA), Rua Sarmento Leite, 245, Porto Alegre 90050-170, RS, Brazil; giulianor@ufcspa.edu.br; 5 Department of Pathology and Legal Medicine, Federal University of Health Sciences of Porto Alegre (UFCSPA), Rua Sarmento Leite, 245, Porto Alegre 90050-170, RS, Brazil; 6 Laboratory of Genetic Toxicology, Federal University of Health Sciences of Porto Alegre, Rua Sarmento Leite, 245, Porto Alegre 90050-170, RS, Brazil; jenifers@ufcspa.edu.br

**Keywords:** breast cancer, hypermethylation, DNA repair genes, *BRCA1*, recurrence

## Abstract

**Background**: Breast cancer is the most prevalent malignant disease among women. Here, we investigate whether there is an association between disease recurrence in breast cancer patients and the quantitative methylation pattern of seven genes of different DNA repair pathways. **Methods**: Clinical and pathological data from 30 patients treated for sporadic breast cancer were selected according to the following inclusion criteria: follow-up of 5 years, adjuvant chemotherapy and recurrence. Histopathology was verified, and genomic DNA was accessed by tumor cryosectioning. We also determined the methylation levels of seven DNA repair genes (*BRCA1*, *BRCA2*, *XRCC1*, *PARP1*, *ERCC4*, *MGMT*, and *XPC*). **Results**: Patients without recurrence demonstrated a higher index of positive progesterone receptor status compared to patients with recurrence (*p* = 0.025). All other clinical characteristics of the patients did not differ between the groups. *BRCA1* and *BRCA2* genes showed methylation, and there was a higher level of *BRCA1* gene methylation in patients without recurrence. *BRCA1* methylation was not associated with the clinical characteristics of patients. All other genes analyzed showed no difference in methylation between patients with and without recurrence. **Conclusions**: We showed that sporadic breast cancer patients with a lower incidence of recurrence demonstrate a higher level of BRCA1 gene methylation after 5 years of follow-up, suggesting its role as a predictive biomarker.

## 1. Introduction

Breast cancer represents the most frequent cancer in women worldwide [1]. In 2024, 310,720 new cases and 42,250 deaths were predicted in the United States [2]. In Brazil, there is an estimate of 73,610 new cases per year, while in the south of the country there is an estimated risk of 71.44 cases per 100,000 women [3]. Breast cancer is heterogeneous in pathological processes, prognosis, and treatment response [4,5]. For example, breast cancer is intimately related to unrepaired DNA damage and deregulation of DNA repair pathways [6,7,8], such as alterations in genes related to DNA repair pathways (e.g., single-strand breaks (SSBs) and double-strand breaks (DSBs), O^6^-alkyguanine, interstrand DNA cross-links, base adducts, insertion–deletion) that play a role in genome stability [6,9]. In addition, homologous recombination (HR) is considered a high-fidelity and conservative repair mechanism of DNA double-strand breaks (DSBs) [10,11]. Efficient HR repair requires functional involvement of breast cancer susceptibility genes (BRCA1 and BRCA2). For instance, BRCA1 appears to play a crucial role as a DNA damage checkpoint activator and promotor of HR, whereas BRCA2 is a core component of HR [11,12]. In addition, methylation of BRCA1 has been associated with an increased risk of breast cancer and specific clinical characteristics of the disease [13]. In the British population, the genetic alteration c.-107 A/T has been shown to cause allelic methylation, leading to familial breast cancer; however, this variant has not been detected in the Polish population [14].

Studies have demonstrated that genomic mutations and epigenetic events in genes involved in DNA repair can also drive breast carcinogenesis [9,15,16,17]. DNA methylation is one of the most frequent epigenetic alterations observed in breast cancer, and consequently it has been considered a potential biomarker of disease progression and treatment response [18,19,20]. However, the mechanism of drug resistance is still poorly understood and predicted by conventional clinicopathologic parameters. For example, drug resistance can be mediated by epigenetic alterations like chromatin modification and hypermethylation of CpG islands [21], as demonstrated by the change in the methylation pattern of diverse genes against tamoxifen and fulvestrant resistance [22,23], as well as the higher responsiveness to cisplatin observed following the epigenetic inactivation of the BRCA1 CpG island promoter in breast cancer cell lines [24]. Thus, here, we investigate whether there is an association between disease recurrence in breast cancer patients and the methylation quantitative pattern of seven genes of different DNA repair pathways (including BRCA1 and BRCA2 of HR; MGMT of direct repair; XRCC1 and PARP1, representing base excision repair; and XPC and ERCC4 of nucleotide excision repair).

## 2. Methods

### 2.1. Sample Collection

A retrospective cohort of 398 breast cancer patients from Femina’s Hospital Mastology Service (Porto Alegre, RS, Brazil) was reviewed to select a group of patients with sporadic breast cancer according to the following inclusion criteria: follow-up of 5 years, adjuvant chemotherapy, and recurrence. The exclusion criterion was neoadjuvant chemotherapy. As a result, 30 patients were enrolled in the study: 15 cases with disease recurrence and 15 without recurrence after five years of follow-up. Surgically removed and confirmed tumor tissues were collected and stored at −80 °C until subsequent procedures. All patients were administered adjuvant systemic therapy, scheme docetaxel, doxorubicin and cyclophosphamide (ACT) or fluoracil, doxorubicin and cyclophosphamide (FAC). After obtaining written informed consent from all participants, complete clinical data of the patients were collected. The study was approved by Ethical Committee of the Federal University of Health Sciences of Porto Alegre (Ref. 10-641) on 31 August 2018. The sample size in the study, necessary to obtain reliable data, aligns with similar pilot methylation studies designed to generate preliminary data [25,26].

### 2.2. DNA Extraction and Quality Check

Before DNA extraction, a section of tumor tissue was obtained by cryosectioning, and tumoral area was confirmed by histopathology. Genomic DNA of breast tumor samples was isolated using PureLink™ Genomic DNA Mini Kit (Invitrogen™, Carlsbad, CA, USA). The quality and quantity of DNA were determined using agarose gel and NanoSpec (Shimadzu, Kyoto, Japan).

### 2.3. Determination of Methylation Status Using EpiTect Methyl II PCR System

Analysis of Promoter CpG island methylation was performed with Qiagen EpiTect Methyl II System (Qiagen, Hilden, Germany) according to the manufacturer’s protocol. A total of 1 µg Genomic DNA from each sample was cleavage using EpiTect^®^ Methyl II DNA Restriction Kit (Qiagen, Hilden, Germany). Four equal aliquots portions of genomic DNA were subjected to Mock (no enzyme), methylation-sensitive (MSRE), methylation-dependent (MDRE), and double (MSRE and MDRE) restriction endonuclease digestion. Afterwards, the aliquots were submitted to real-time PCR using EpiTect Methyl PCR Assay (Qiagen, Hilden, Germany) primers targeting promoter CpG islands *BRCA1* (CpG Island 106038-Chr17: 41278134-41278460); *BRCA2* (CpG Island 103696-Chr13: 32889533–32889900); *XRCC1* (CpG Island 107552-Chr19: 44079582–44079905); *PARP* (CpGIsland 101327-Chr1: 226595333–226595939); *ERCC4* (CpGIsland 105086-Chr16: 14013751–14014295); *XPC* (CpGIsland 110069-Chr3: 14219715–14220408); and *MGMT* (CpGIsland 102002-Chr10: 131264948–13125768), with RT^2^SYBR Green qPCR Mastermix (Applied Biosystems, Carlsbad, CA, USA), in the StepOnePlus instrument (Applied Biosystems, Carlsbad, CA, USA). This step allows for the quantitative detection of remaining DNA after digestion with MSRE and MDRE restriction enzymes. Two controls were used to test to digestion efficiencies of the enzymes (EpiTect Methyl II PCR Assay EP_SEC and EP_DEC, Qiagen, Hilden, Germany) [27,28]. For each gene in each sample, we calculated the methylation status as the percentage of methylated and unmethylated DNA. When ΔCt (MSRE−Mock) ≥ 4 for SEC or ΔCt (MDRE−Mock) ≥ 4 for DEC, this indicates that more than 93.8% of control DNA molecules were digested, confirming that the restriction enzyme is active and digests DNA efficiently [27]. The methylation level of individual genes (%) was calculated from CT values, with the EpiTect Methyl II PCR Array and Microsoft Excel. A cut-off value of 10% methylation was adopted to define elevated methylation levels, as previous described in [29].

### 2.4. Statistical Analysis

The associations between variables were analyzed by χ^2^ tests. For comparing continuous variables, a Student’s *t* test or an unequal variance *t*-test was used. Kaplan–Meier plots for cumulative survival were compared using log rank tests. In all analyses, *p*-values were set at <0.05. All data were analyzed using the Statistical Package (PASW Statistics for Windows, Version 18.0. SPSS Inc., Chicago, IL, USA).

## 3. Results

**Figure 1:**
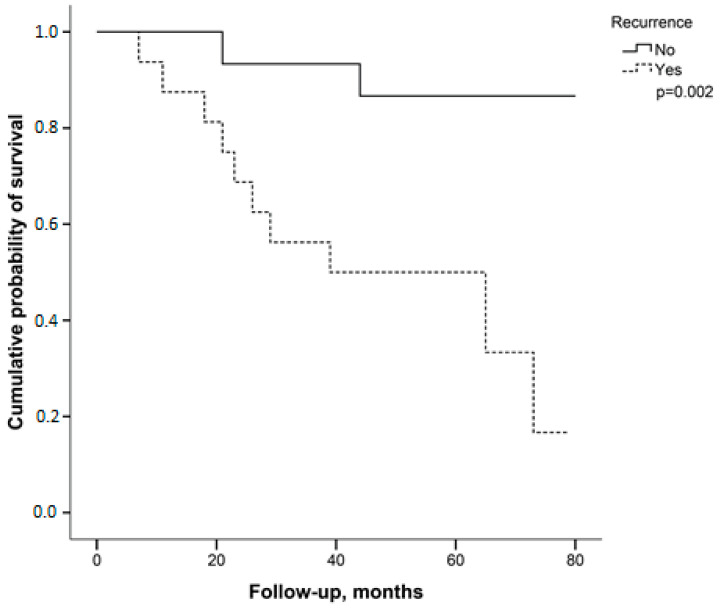
Kaplan–Meier curve of follow-up for breast cancer patients stratified by recurrence status.

Patients without recurrence demonstrated a higher index of positive progesterone receptor (PR) status compared to patients with recurrence (*p* = 0.025). All other clinical characteristics of the patients did not differ between the groups (Table 1). *BRCA1* and *BRCA2* genes showed methylation (Table 2). There were higher levels of *BRCA1* gene methylation in patients without recurrence (*p* = 0.012). *BRCA1* methylation was not associated with the clinical characteristics of patients. All other genes analyzed showed no difference in methylation between patients with and without recurrence (Table 2). Patients with recurrence showed higher risk of death than patients without recurrence (*p* < 0.05, Figure 1).

## 4. Discussion

We showed that sporadic breast cancer patients with a lower incidence of recurrence demonstrate higher levels of BRCA1 gene methylation, after 5 years follow-up. These findings reinforce that higher levels of *BRCA1* gene methylation can be a protective factor to disease recurrence [30]. However, the role of BRCA1 methylation concerning sporadic tumors is still unclear. For example, hypermethylation of the BRCA1 promoter region contributes to disease relapse and progression by mimicking the consequences of germline BRCA1 mutations, leading to gene silencing and preventing effective HR repair [13]. On the other hand, patients who underwent adjuvant chemotherapy with *BRCA1*-mutated and *BRCA1*-hypermethylated tumors exhibited better survival compared to those with *BRCA1*-proficient tumors [30]. Therefore, gene silencing and HR deficiency could lead to a higher responsiveness to platinum-based therapy (a protective factor) [31]. Hypermethylation may also be an early event in tumor development that progresses along a common pathway to the *BRCA1*-mutated disease, representing a promising DNA-based biomarker for early-stage TNBCs [32]. In addition, the BRCA1 promoter methylation pattern differs across tumors and tissue, as it is higher in malignant breast tumors and normal adjacent tissues (NATs) compared to benign breast lesions [33]. These findings suggest that BRCA1 promoter hypermethylation is a potential useful biomarker of aggressiveness in breast tumors; in addition, both mutation and methylation improve the response to DNA-damaging chemotherapy by impairing DNA repair. In line with this, a better survival rate was observed in early-onset TNBC patients with impaired BRCA1 treated with ACT chemotherapy, suggesting that the better responsiveness to treatment may be due to a defective HR pathway [30].

In addition, in cancer cell lines and xenografted tumors, *BRCA1* CpG island promoter hypermethylation is associated with sensitivity to platinum-derived drugs in the same fashion as *BRCA1* mutation [24]. BRCA promotor hypermethylation seems to be a rare event in metastatic BC but is preserved in subsequent xenograft models and might represent an attractive therapeutic marker for PARP inhibitors (PARPis) [31,34]. In addition, quantitative BRCA1 promoter methylation assessment might predict treatment response in PARPi and analysis of BRCA1/2 methylation in liquid biopsies might determine patient subgroups at different time points that may benefit from PARPis [34]. For example, in ovarian cancer patients treated with cisplatin, *BRCA1* epigenetic inactivation was associated with a significantly longer time to relapse and improved overall survival [24]. The human cell lines with epigenetically silenced *BRCA1* through gene hypermethylation exhibited sensitivity to PARP inhibitors, as did the *BRCA1* mutation [35].

Furthermore, several studies have reported associations between DNA repair methylation genes and the clinical characteristics of breast cancer patients [30,36,37,38]. For instance, *BRCA1* promoter hypermethylation is associated with tumors with a more aggressive clinical–biological profile [39]. Thus, the determination of *BRCA1* promoter methylation status could enable adjustments of antitumor therapy for patients [40]. Moreover, *MGMT* promoter methylation is also associated with breast cancer risk and negative expression of the MGMT protein [37,41]. An association between *BRCA1* promoter methylation and significantly worse disease-free survival (mainly in TNBC patients [30,38]) has also been demonstrated [42,43]. The *BRCA1* deficiency (including the pathogenic variant in the *BRCA1* gene or *BRCA1* promoter hypermethylation) is recurrent in early-onset TNBC and is related with enhanced survival [30]. However, in our study, we found no association between gene methylation levels (*BRCA2*, *XRCC1*, *PARP*, *ERCC4*, *XPC*, *MGMT*) and disease recurrence in breast cancer patients. In contrast, other tumors, such as in gastric cancer, hypermethylation of *XRCC1* has been demonstrated, as well as hypermethylation of *XPC* in bladder and lung cancer [44,45,46].

We also demonstrated that 80% of patients without recurrence were positive for the progesterone receptor, which can be associated with prolonged BRCA1 promoter hypermethylation maintenance, which leads to the remodulation of hormone receptors shortly after the induction of the BRCA1 promoter hypermethylation decreases PR levels [47]. In addition, PR status is a well-established prognostic factor in breast cancer [48]. Finally, our study presents some limitations and potential lines for further research. For example, the inclusion of only patients submitted to surgery without neoadjuvant chemotherapy could contribute to a better understanding of gene hypermethylation and breast cancer, as could analyzing other genes and samples from different populations. The sample size of our study can also be considered a limitation (e.g., our sample size does not allow for performing a multivariate analysis with high statistical power). Another limitation is the evaluation of the single CpG island, through EpiTect Methyll II PCR assays, that was available for each candidate gene promoter region. We did not evaluate the methylation status of CpG islands located in gene bodies or the transcription start sites, nor did we evaluate the potential influence that DNA sequence variation may have on the methylation status of neighboring CpGs, even though CpGs are most commonly found in gene promoters. Taken together, our findings suggest that higher levels of BRCA1 gene methylation are associated with the absence of recurrence, indicating that the lack of a functional BRCA1 contributes to chemosensitivity in breast cancer. These findings reinforce the importance of measuring methylation levels in BC patients to enable clinical decision making.

## Figures and Tables

**Table 1:** Clinical characteristics of the study baseline.

Endpoints	N = 30	Recurrence		*P*
		Without (*n* = 15)	With (*n* = 15)	
Age, years	53.70 ± 12.17	54.40 ± 11.02	53.00 ± 13.57	0.759
Tumor size, cm	3.59 ± 2.46	3.57 ± 2.20	3.61 ± 2.76	0.775
Tumor grade				0.335
I	1 (3.3)	1 (6.7)	0	
II	15 (50.0)	8 (53.3)	7 (46.7)	
III	14 (46.7)	6 (40.0)	8 (53.3)	
TNM stage				0.066
I-II	14 (46.7)	10 (66.7)	4 (26.7)	
III	16 (53.3)	5 (33.3)	11 (73.3)	
Vascular invasion				0.218
No	8 (30.8)	5 (45.5)	3 (20.0)	
Yes	18 (69.2)	6 (54.5)	12 (80.0)	
Unknown	4	4		
Biological subtype				0.367
Luminal A	6 (20.7)	3 (21.4)	3 (20.0)	
Luminal B	13 (44.8)	8 (57.1)	5 (33.3)	
HER2+	2 (6.9)	0	2 (13.3)	
Triple negative	8 (27.6)	3 (21.4)	5 (33.3)	
Unknown	1	1		
Estrogen receptor				0.245
Negative	10 (33.3)	3 (20.0)	7 (46.7)	
Positive	20 (66.7)	12 (80.0)	8 (53.3)	
Progesterone receptor				
Negative	13 (43.3)	3 (20.0)	10 (66.7)	0.025
Positive	17 (56.7)	12 (80.0)	5 (33.3)	
Ki67				0.700
14%<	10 (34.5)	4 (28.6)	6 (40.0)	
>14%	19 (65.5)	10 (71.4)	9 (60.0)	
Unknown	1	1		
Radiotherapy	27 (90.0)	14 (93.3)	13 (86.7)	1.000
Hormone therapy	19 (67.9)	11 (73.3)	8 (61.5)	0.689
Follow-up, months	50.57 ± 23.80	60.60 ± 19.51	40.53 ± 24.03	
Death	12 (40.0)	2 (13.3)	10 (66.7)	

Note: Data are presented as No. (%) or mean ± SD (standard deviation). Patients with unknown characteristics were not included in *p* value calculations; therefore, percentages for these groups are not depicted in the table.

**Table 2:** Comparisons between the level of gene methylation and disease recurrence in breast cancer subjects.

Genes	*n* = 30 (%)	Recurrence (%)		*P*
		Without (*n* = 15)	With (*n* = 15)	
*BRCA1*	60.30 ± 23.60	69.91 ± 14.96	49.44 ± 24.99	0.012
*BRCA2*	12.83 ± 28.27	4.21 ± 12.77	14.85 ± 29.65	0.838
*XRCC1*	03.43 ± 18.22	0.08 ± 0.141	0.12 ± 0.283	0.345
*PARP*	05.34 ± 20.64	3.94 ± 14.66	0.09 ± 0.169	0.967
*ERCC4*	09.12 ± 25.98	6.79 ± 23.09	0.67 ± 0.898	0.345
*XPC*	03.72 ± 18.12	0.39 ± 0.610	0.41 ± 0.571	0.653
*MGMT*	09.90 ± 25.28	4.34 ± 15.43	5.36 ± 17.97	0.539

Note: Data are presented as means ±SD.

## Data Availability

The data presented in this study are available on request from the corresponding author (data are not publicly available due to ethical approval restrictions).
